# Supplementary material for: First Insights about Antiparasitic and Action Mechanisms of the Antimicrobial Peptide Hepcidin from Salmonids against Caligus rogercresseyi
Source: Pharmaceutics. 2024 Mar 8;16(3):378. doi: 10.3390/pharmaceutics16030378 (PMC10975781; doi:10.3390/pharmaceutics16030378)
Supplement: Supplementary file 1 [file pharmaceutics-16-00378-s001.zip › pharmaceutics-2866708-supplementary.pdf]

Table S1: Structure model prediction of salmonid antimicrobial peptides.

Peptide	Residue prediction*	3D model
**GsD**	Sequence FIGGIISFFKRLF<br>Prediction CCCHHHHHHHHHC	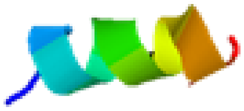
**H2B**	Sequence VSEGTHAVTKYTSSK<br>Prediction CCCCCSSSSSSSCCC	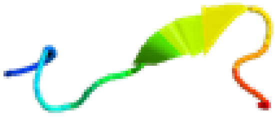
**Epinecidin**	Sequence GFIFHIIKGLFHAGKMIHGLV<br>Prediction CCSSHHHHHHHHCCHHHHCCC	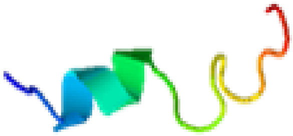
**Piscidin**	Sequence FFHHIFRGIVHVGKTIHKLVTG<br>Prediction CHHHHHHHHHHCCHHHHHHHCC	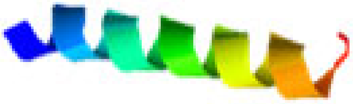
**Hepcidin**	Sequence LCRWCCNCCHNKGCGFCCKF<br>Prediction CCHHHHCCCCCCCCCSSSCC	Álvarez et al., 2014 (27)

* The structural motifs are indicated as H, S, and C for α-helix, β-sheet, and random coil, respectively.

Table S2. Mass and purity of synthetic peptides

<table>
<tr><th>Peptide</th><th>HPLC spectrum</th><th>ESI-MS Mass spectrum</th></tr>
<tr><td>GsD</td><td>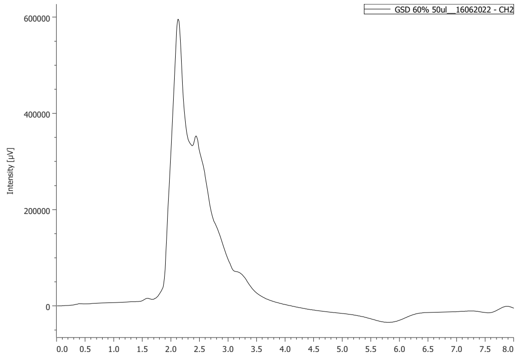
</td><td>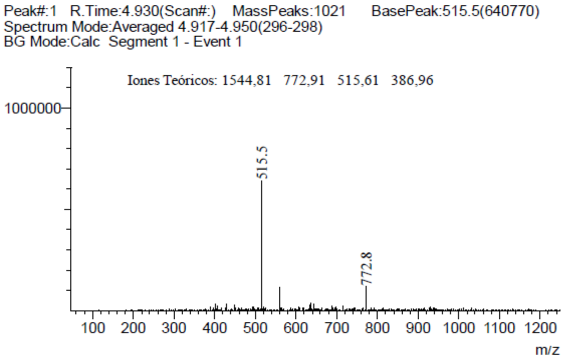
</td></tr>
<tr><td>H2B</td><td>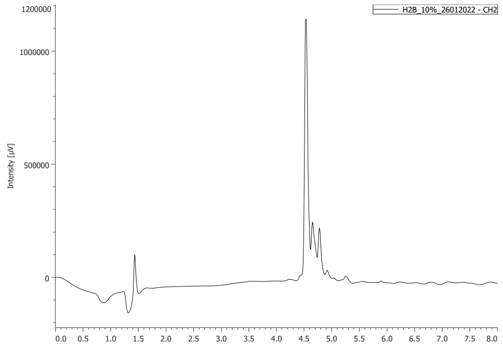
</td><td>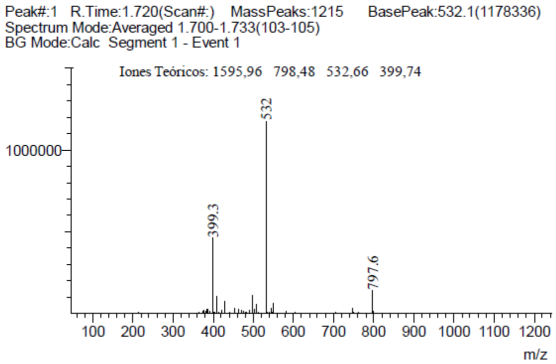
</td></tr>
<tr><td>Epinecidin</td><td>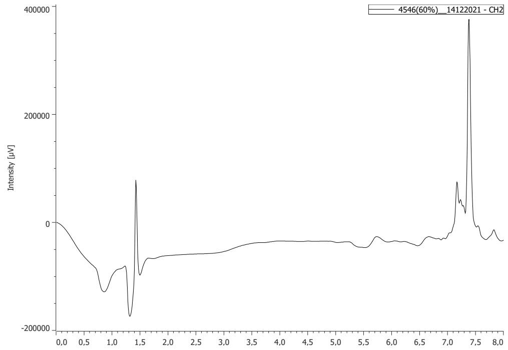
</td><td>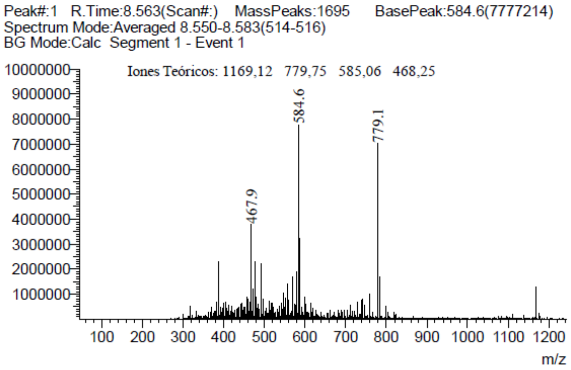
</td></tr>
<tr><td>Piscidin</td><td>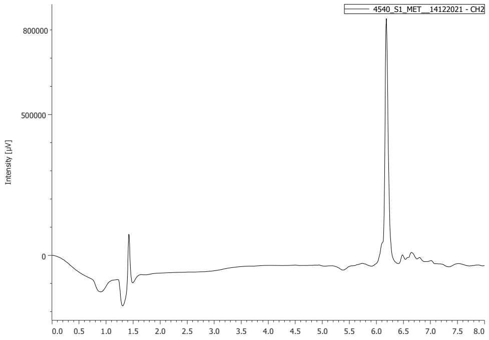
</td><td>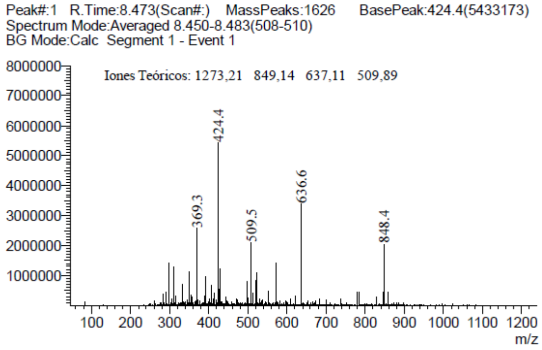
</td></tr>
<tr><td>Hepcidin</td><td>Álvarez et al., 2014 (27)</td><td>Álvarez et al., 2014 (27)</td></tr>
</table>
